# Exploring the forced circumstances leading to early sexual debut among primary school learners in Musina, Limpopo

**DOI:** 10.1177/22799036251388581

**Published:** 2026-04-22

**Authors:** Ikekhwa Albert Ikhile, Azwihangwisi Helen Mavhandu-Mudzusi, Ayobami Precious Adekola, Vhothusa Edward Matahela, Hulisani Matakanye, Livhuwani Tshivhase

**Affiliations:** 1 Department of Gender and Sexuality Studies, College of Human Sciences, University of South Africa, Pretoria, South Africa; 2 College of Human Sciences, University of South Africa, Pretoria, South Africa; 3 Department of Health Studies, College of Human Sciences, University of South Africa, Pretoria, South Africa

**Keywords:** early sexual behaviour, primary school learners, musina, structural violence, gender dynamics, peer pressure, sexual health education, socioeconomic factors

## Abstract

**Introduction:**

This study explored the forced circumstances contributing to early sexual debut among primary school learners in Musina, Limpopo, focussing on the socio-economic, cultural, and educational factors shaping this phenomenon.

**Methods:**

A qualitative cross-sectional design was adopted, involving 320 learners (Grades 5–7) and 20 Life Orientation teachers from eight schools. Data collection for learners involved classroom-based group discussions conducted in a developmentally appropriate manner, while teachers participated in a separate workshop to provide contextual insights on learner experiences. Thematic content analysis was conducted using Bronfenbrenner’s social-ecological model, Ajzen’s theory of planned behaviour, and Galtung’s structural violence framework.

**Results:**

Learners reported that early sexual debut was primarily influenced by forced circumstances such as poverty, lack of basic needs, coercion, and peer pressure, rather than voluntary risky behaviours. Cultural expectations, familial pressures, and gender power imbalances further constrained their agency. Teachers highlighted barriers, including insufficient training and socio-cultural taboos, that limited their ability to support learners.

**Conclusion:**

Early sexual debut among primary school learners in Musina is largely a result of external forced circumstances occurring outside the school environment, particularly linked to socio-economic deprivation and coercive pressures. Addressing this requires public health interventions that are multi-level and contextually tailored, including socio-economic support for vulnerable families, community-based sexual health education, and policies that protect children from exploitation and coercion.

## Introduction

Adolescent sexual debut and its related behaviours, especially in rural areas, is of considerable concern to global health agencies today.^1^ Research has shown that in South Africa, a large number of adolescents have had a sexual experience before reaching 15 years of age.^2^ The rural areas of Limpopo province, such as the Vhembe District, face particular problems relating to early sexual behaviour among primary school learners.^3^ However, there is a scarcity of context-specific studies focussing on early sexual debut in rural South Africa, highlighting a significant research gap. This study aims to fill this gap by investigating the forced circumstances leading to early sexual debut among primary school learners in Musina, Limpopo. It is, therefore, important to have an understanding of the local context if one is to identify the socioeconomic, cultural, and educational factors that give rise to early sexual debut in such settings. This mirrors observations made by other researchers that suggest that early sexual activity is an outcome of factors other than affection for the sexual partner.^4,5^

The theoretical frameworks guiding this study include the social-ecological model, which examines the interplay between individual, relationship, community, and societal factors; the theory of planned behaviour, which focuses on individual attitudes, subjective norms, and perceived behavioural control; and the concept of structural violence, which refers to systematic ways in which social structures harm or disadvantage individuals. These frameworks collectively provide a comprehensive lens to understand the multifaceted drivers of early sexual debut in this context.

Socio-economic factors have been shown to have a profound effect on the sexual activity of adolescents, with Madise et al.^6^ having identified access to few or no resources and poor economic status as contributing to adolescents’ engagement in risky behaviours. The situation is, however, further exacerbated by cultural beliefs and practices in rural South African settings that could also influence the timing of the first sexual experience.^7,8^ Comprehensive sex education has been found to reduce risky sexual behaviour among school learners.^9^ However, the impact of such programmes in the rural South African context is questionable, as some studies have found a disconnect between points of teaching in the curriculum and the learners’ contexts.^10^

Our study is one of the components of a larger community-engaged scholarship project in the Soutpansberg North Circuit of Limpopo Province, intending to investigate the factors contributing to early sexual behaviour among primary school learners in Musina, Vhembe District. Through the adoption of a qualitative research strategy, we aimed to provide a better understanding of the views of both learners and teachers and, as a result, to shed more light on what we considered to be a topical issue.

### Geographical study area

The study area selected for the research was Musina, a rural community in the Vhembe District of Limpopo province in South Africa. Due to its rural nature, Vhembe District has poor socio-economic growth.^11^ Musina is the northernmost town in Limpopo province and lies on the Limpopo River across the border with Zimbabwe. Its proximity to the Beit Bridge border point and its location along the N1 highway make it an important point of access to the country.^12^ Its geographical situation exposes the town to cultural influences and economic activities such as long-haul truck traffic and cross-border business.^13^ Factors that negatively affect the economy include a semi-arid climate, and historically, the town has relied primarily on the mining business.^14^ These factors, in combination, determine the socio-economic context within which young people experience their sexual development and make sexual decisions.^15^

Like many other rural areas in South Africa, Musina experiences problems such as high unemployment, limited access to health care, and a lack of adequate educational resources. Furthermore, a relatively large percentage of Musina’s population is below 18.^11^ Learners in the primary schools, the target group of our study, are between the ages of 6 and 13. The community is mainly Tshivenda-speaking and culturally rooted, with values and practices that are still somewhat traditional while simultaneously being affected by current social problems.^16^

### A powerful statement

During the group discussions that we held with primary school learners in Musina, one of the participants made a powerful statement that, in effect, sums up the reasons why primary school learners in the study area engage in sexual activity at this early age: “It is not that we love sex but because the circumstances compel us.” This suggests that these young learners feel a lack of agency when agreeing to participate in sexual activities; they are propelled into having sex by forces that are beyond their control. We hope that the inclusion of this statement in our article may help to change perceptions about early sexual debut in rural South African settings, as it differs from the simplistic views of adolescent sexuality that are often expressed and instead calls for increased attention to the socio-economic, cultural and structural contexts that inform adolescents’ sexual practices.^17^

### Research objectives and questions

We felt it important to include the above statement early on in this paper, in which we report on our investigation into the forced circumstances in which the learners to whom we spoke found themselves. Our study was guided by the following three main objectives, each of which we hoped to achieve through finding answers to specific research questions:

By obtaining responses to the questions in Table 1, we aimed to help develop a better understanding of early sexual behaviour among primary school learners in Musina. It is hoped that the study’s results will guide the design of appropriate prevention and policy measures that may benefit comparable rural areas in South Africa and other countries.

**Table 1.** Research objectives aligned with research questions.

Research objective	Research question
To understand the socio-economic, cultural, and educational factors that influence early sexual debut among primary school learners in Musina.	What socio-economic factors in Musina propel learners towards early sexual behaviour?
	How do cultural norms and practices in the community affect learners’ sexual activities?
	How does the current education environment influence learners in terms of their perceptions about and participation in sexual activities?
To explore the perceptions and experiences of learners in relation to early sexual awareness and behaviour as a result of forced circumstances.	How do learners understand and express the meanings of the expression “forced circumstances”?
	What specific pressures or influences do learners identify as being behind their involvement in various sexual activities?
	To what extent do learners feel they have control over their decisions regarding sexual behaviour?
To assess the capacity and challenges educators face in delivering effective sex education and support to learners.	What knowledge and resources do educators currently have for delivering sex education?
	What barriers do educators face in addressing early sexual behaviour among their students?
	How do educators perceive their role in mitigating the “forced circumstances” described by learners?

## Literature review

### Early sexual debut in South Africa: Prevalence and trends

Early sexual debut among adolescents is a serious public health concern in South Africa owing to its repercussions for sexual health and education. Several studies have shown socio-economic phenomena and urbanisation to play a role in early sexual activity among adolescents, with adolescents in urban areas at a higher risk due to social influences.^18^ For instance, it was observed that adolescent girls in urban settings are more likely to become sexually active early on as a result of peer pressure and media.^19^ Another study found there to be a positive relationship between educational level and the risk of early sexual debut; therefore, improved education could be vital in preventing this.^20^ National surveys, such as the South African Demographic and Health Survey, estimate that approximately one in three adolescents engage in sexual activity before the age of 15, with prevalence rates particularly high in rural provinces like Limpopo and KwaZulu-Natal.^11^ Regional comparisons also show variation, with studies from Zimbabwe and Mozambique reporting similar patterns of early sexual initiation linked to poverty and limited access to health services.^11,15^

### Factors influencing early sexual behaviour in rural areas

There are several reasons why early sexual behaviour is prevalent in rural areas, and these include a lack of adequate information on sexuality, cultural beliefs and practices, and economic factors.^21^ In rural areas, there is limited access to comprehensive and accurate sex education, and therefore, many people lack accurate information on the subject.^20^ The influence of culture and pressure from peers can intensify the problem since people, adolescents particularly, may feel pressured to conform to current trends concerning sexual relations.^22^ The research examining the early sexual experiences of adolescents in South Africa further documented high levels of coercion and violence during first-time sexual experiences, making the overall picture of adolescent early sexual behaviour even bleaker.^22^ This suggests that more age-appropriate and focussed strategies to correct the educational gaps and consider socio-cultural factors are warranted.

### Impact of early sexual activity on education and health

The adverse effects of early sexual activity are not restricted to health but also negatively affect the education and well-being of children. Studies have found that early engagement in sexual activity increases the likelihood of sexually transmitted infections (STIs) and pregnancy, especially in the case of adolescents who are not in a mutually monogamous relationship that is free of violence and does not include an exchange of resources.^23^ Pregnancy and the health risks mentioned above can result in adolescents dropping out of school, bringing an end to their education; this then perpetuates the cycle of socio-economic disadvantage.^24^ In addition, the stigma attached to adolescent sexual behaviour increases the chances of complications arising from the early initiation of sexual activity, since the adolescent cannot easily access the necessary health services.^25^ The connexion between health and education indicates the need for synergistic models that incorporate both elements. Further evidence from Kenya, Uganda, and Nigeria confirms similar outcomes, with early sexual initiation linked to increased dropout rates, psychological distress, and long-term economic vulnerabilities.^5,19,26^

### Interventions and policies

In this section, we present some of the interventions and policies that have been introduced relating to early engagement in sexual behaviour among adolescents. School-based interventions seeking to increase adolescents’ knowledge about sex and promote self-efficacy have been effective in decreasing risky behaviour.^19^ For example, interventions such as “You Only Live Once” have successfully promoted positive behaviour change and enhanced mediating factors relating to sexual health among South African adolescents.^27^ Topics such as consent, healthy relationships, and the negative effects of early engagement in sexual activity for women should be taught within comprehensive sex education (CSE) to enable adolescents to make better decisions.^28^ However, these interventions are only as effective as the “boots on the ground” that support them and the engagement of family members in the decision to deal with matters of adolescent sexual health.^28^ Other noteworthy interventions include South Africa’s “Rise Young Women’s Clubs” and “Soul Buddyz Club,” both of which have been shown to enhance life skills and delay sexual debut among school-going adolescents.^12,17^ In addition, regional programmes such as DREAMS (Determined, Resilient, Empowered, AIDS-free, Mentored, and Safe) in several African countries have demonstrated success in addressing structural drivers of early sexual debut.^17^ These interventions highlight the need for multi-level approaches that address not just individual behaviour but also socio-economic vulnerabilities.

### Recent trends and global practices

Recent studies have highlighted a concerning decline in condom use among adolescents. A World Health Organization (WHO) report covering data from 2014 to 2022 across 40 countries, including those in Europe and Canada, indicates that condom usage during the last sexual encounter decreased from 70% to 61% in boys and from 63% to 57% in girls aged 15. This decline underscores the need for enhanced sexual education and access to contraceptive methods to mitigate risks associated with unprotected sex.^29^

In response to such trends, global best practices in comprehensive sexuality education (CSE) have evolved. A UNESCO global status report emphasises the importance of implementing age appropriate CSE that covers topics like contraception, sexually transmitted infections (STIs), consent, and gender equality. The report recommends involving communities, including parents and religious leaders, to create a supportive environment for CSE and highlights the necessity of training teachers to deliver these programmes confidently.^30^

Additionally, innovative approaches to sex education are being adopted globally. For instance, France has introduced a curriculum aimed at fostering gender equality and teaching concepts of consent starting from an early age. This programme educates 4-year-olds about gender equality, scientific terms for genitalia, and the concept of consent, while older students learn to differentiate sex, gender, and sexual orientation. ^31^ Similarly, Canada and Sweden have implemented nationwide CSE frameworks that integrate digital literacy and address online sexual risks, while countries such as Rwanda and Ethiopia have piloted community-based models that engage parents and adolescents together.^29,30^ These diverse global practices provide important lessons for tailoring interventions in the South African rural context.

Incorporating these global insights and addressing recent trends is crucial for developing effective interventions tailored to the South African context. By learning from international best practices and understanding current challenges, stakeholders can enhance the effectiveness of programmes aimed at reducing early sexual debut and promoting sexual health among adolescents.

Resolving the problems attendant on early sexual behaviour among primary school learners in Musina, Vhembe District, requires a multifaceted approach that takes into account the unique socio-cultural and economic contexts of the region. By enhancing educational opportunities, providing comprehensive sexual health education, and fostering supportive community environments, it is possible to mitigate the risks associated with early sexual initiation and promote healthier outcomes for adolescents.

## Methodology

### Theoretical framework

This study employed an integrated theoretical framework combining Bronfenbrenner’s Social-Ecological Model, Ajzen’s Theory of Planned Behaviour (TPB), and Galtung’s Structural Violence Theory to examine the complex factors influencing early sexual debut among primary school learners in Musina. Bronfenbrenner’s^32^ model provided a multi-layered lens to explore how environmental systems, including family (microsystem), peer and school relationships (mesosystem), and broader socio-cultural conditions (macrosystem), shape sexual behaviours. This framework contextualised learners’ exposure to sexual activity within overlapping domestic, educational, and community spaces.^33^ Ajzen’s^34^ was applied to understand how learners’ intentions were shaped by attitudes (e.g. material motives), subjective norms (e.g. peer expectations), and perceived behavioural control (e.g. economic vulnerability and knowledge gaps). The model elucidated how these psychological factors interact with learners’ constrained environments, influencing their decisions. Galtung’s^35^ concept of structural violence was used to interrogate how systemic inequalities, poverty, gender disparities, and lack of education limit learners’ choices and normalise risky sexual behaviour. These structural forces compel engagement in exploitative relationships, often under the guise of necessity.^36^ Together, these frameworks offered a holistic analytical approach that moved beyond individual-level explanations, positioning early sexual debut as the outcome of interrelated personal, cultural, and structural conditions. This integrated model strengthened the study’s capacity to inform contextually grounded, multi-level interventions targeting adolescent sexual health.

### Research design

For the purposes of the study, a cyclical longitudinal ethnography design was used to explore the reasons for early engagement in sexual behaviour among learners attending primary school in Musina, South Africa. Through the ethnographic approach, a detailed investigation of social phenomena over time is possible, along with recording rich contexts and patterns in the learners’ experiences and behaviours.^37^ The repetitive structure of the design enabled the researchers to collect data and carry out analysis continuously, which improved their insight into their subject. This study is part of a long-term community engagement project that commenced in 2012 and is still ongoing. As part of this initiative, the research team visits the study site annually for a 1-week period to collect data while supporting and monitoring the implementation of sexual health and educational interventions in an iterative and participatory manner.

### Study sample

The sample comprised 320 learners, of whom 164 were female and 156 were male, from eight primary schools in Musina. The participants were in Grades 5–7, with an age range of 10–16. Grades 5–7 were specifically chosen because this age range is critical for the formation of sexual awareness and behaviours among adolescents. The selection of these grades aligns with the research objective to understand early sexual engagement during this formative period. Schools were selected based on their geographic location within Musina and willingness to participate, ensuring a representative sample of the local educational environment. The representation and range within the sample ensured the presentation of a broad spectrum of experiences and views that enhanced the quality and authenticity of the data.^38^ Within each school, learners were further divided into smaller discussion groups of 6–8 participants to enable more meaningful engagement and reduce peer-influence dominance. Exclusion criteria included learners below the age of 10, learners not enrolled in Grades 5–7, and those whose parents or guardians declined consent or who themselves refused assent. Learners with known cognitive or developmental challenges that could impair participation in group discussions were also excluded, in line with ethical guidelines.

### Data collection methods

Data collection involved mostly group discussions with learners in the classroom context. This method was adopted to create a friendly space, allowing participants to share their thoughts and experiences easily. In teen research, group discussions often prove effective, as they support open communication and reduce the power differential between researchers and participants. The group discussions were structured using a semi-structured interview guide, allowing flexibility while ensuring key topics were covered. Sample questions included: “Can you describe what you understand by sexual risk-taking behaviour?” and “What factors do you think influence young people to engage in sexual activities at an early age?” Communication strategies appropriate to the learners’ developmental stage were applied for successful interaction.^39^

In addition to the discussions with the learners, a 3-day workshop was held with Life Skills and Life Orientation teachers from each participating school. The workshops presented the opportunity for data triangulation, consolidating the study through the addition of teachers’ perspectives on the school environment and the obstacles encountered in the management of early engagement in sexual activity among students.^40^ Importantly, learners and teachers were engaged separately to avoid cross-influence of responses, ensuring that learners could speak freely about their experiences while teachers provided broader institutional perspectives.

### Data analysis

Thematic analysis was employed following the reflexive model articulated by Braun and Clarke.^41^ The analytic process included six key phases:

- Familiarisation with the data through reading and re-reading of transcripts,
- Generation of initial codes through manual coding of relevant patterns using both inductive and deductive approaches,
- Searching for themes by collating codes into candidate themes,
- Reviewing themes to ensure internal consistency and alignment with coded extracts and full data set,
- Defining and naming themes based on clarity, conceptual coherence, and relevance to the research objectives, and
- Producing the final report by selecting compelling quotations and interpreting themes within the theoretical frameworks.

To enhance credibility and dependability, the research employed data triangulation from learners and teachers, and peer debriefing was conducted to verify theme coherence. An audit trail was maintained throughout coding and theme development to ensure transparency in analytic decisions. These steps were critical for maintaining analytical rigour and ensuring findings were grounded in participant narratives. In presenting the findings, themes were organised in a hierarchical manner to reflect gradations of experiences. For instance, the overarching theme of “perceived lack of agency (‘forced circumstances’)” was unpacked into sub-themes such as peer pressure, economic necessity, and a sense of inevitability. This gradation provided greater clarity and depth in the thematic representation.

### Sampling strategy

Engagement with learners on this topic made it necessary to include Life Skills and Life Orientation teachers in the study. Our use of purposive sampling ensured that the teachers selected as participants were knowledgeable and actively engaged in these areas, and made it possible to confirm the applicability and extent of their insight.^42^ Their participation was intended to complement, not merge with, the learners’ narratives, acknowledging that teacher perspectives reflect professional observations rather than lived experiences.

### Reflexivity and ethical considerations

Throughout the research process, by maintaining a reflexive perspective, we, as researchers, strove to remain cognisant of our biases and identities. The focus was mainly on ethical aspects, intending to protect adolescent participants’ wellness. We received informed consent from both learners and their guardians, and we took care to maintain the anonymity of the learners and ensure their access to support services in order to prevent excessive stress.

To safeguard participants’ emotional safety while discussing sensitive topics, we ensured all sessions were facilitated by trained moderators familiar with the local context and language. Participants were reminded that they could decline to answer any question or exit the session without repercussions. School-based psychosocial support services were made available throughout the study. The study further acknowledged the sensitivity of discussing forced sexual circumstances with children and therefore prioritised creating non-threatening spaces where learners’ narratives could emerge without fear of reprisal or exposure.

### Ethical approval process

This study received ethical clearance from the College of Human Sciences Research Ethics Committee at the University of South Africa **(Ref: 90187598_SEPTEMBER_CREC_CHS_2023)**. Ethical approval was obtained prior to the commencement of all research activities. The study was conducted in strict adherence to the principles of the Declaration of Helsinki and the South African National Health Research Ethics Council (NHREC) guidelines for research involving minors. Informed written consent was obtained from all participating teachers, while parental/guardian consent was secured in writing for all learner participants. In addition, learners provided verbal and age-appropriate written assent prior to participation, which differed from guardian consent in that learners were asked to confirm their willingness to participate in developmentally suitable language without legal obligations. The research team conducted preliminary classroom engagements to explain the nature, purpose, and expectations of the study in a developmentally appropriate manner, ensuring participants understood their rights, including the right to withdraw at any time without penalty.

To ensure participant safety while discussing sensitive topics related to sexuality, researchers created a supportive, non-judgemental environment during classroom-based group discussions. Trained facilitators, fluent in the local language and culturally competent, used child-sensitive communication strategies to foster trust and emotional comfort. Emotional support services were also arranged in advance, and school counsellors were present or on standby during all sessions. Strict confidentiality and anonymity were maintained throughout the study. No identifying information was recorded or linked to individual responses, and pseudonyms were used during transcription. All qualitative discussions were audio-recorded with participant and guardian permission to ensure accurate data capture. Following transcription and verification of the audio-recorded data, the original recordings were permanently destroyed in accordance with good ethical practice. Data were stored securely in password-protected digital files accessible only to the research team, and physical notes were locked in secure cabinets.

### Triangulation and theoretical framework

The combination of classes with learners and workshops with teachers, guided by a comprehensive qualitative research framework, offered a nuanced and grounded understanding of the elements shaping early engagement in sexual activity among primary school students in Musina.

### Statistical analysis

Given the qualitative nature of this study, data were primarily analysed using thematic content analysis.^41^ Audio-recorded group discussions and workshop sessions were transcribed verbatim and reviewed for accuracy. The transcripts were then coded both manually and with the aid of qualitative data analysis software, NVivo version 12. Coding followed an inductive approach, allowing themes to emerge organically from the data while also being guided by theoretical constructs from Bronfenbrenner’s^32^ social-ecological model, Ajzen’s^34^ theory of planned behaviour, and Galtung’s^35^ structural violence framework.

### Thematic analysis

Based on the theoretical framework proposed by Braun and Clarke,^41^ themes and subthemes were extracted from the data collected during the study using NVivo 12 software. These themes offer a rich understanding of factors that may have an impact on early engagement in sexual activity among primary school learners in Musina and the challenges teachers face in dealing with such topics (Table 2).

**Table 2.** Research themes and sub-themes.

Main themes	Sub-themes
Socio-economic drivers of early engagement in sexual activity	Poverty and material need, lack of alternative activities, transactional relationships
Cultural influences	Traditional practices normalising early sexual experiences, gendered expectations, and taboos relating to sex education
Educational factors	Inadequate sex education, disconnect between curriculum and local realities, teachers’ limited capacity
Perceived lack of agency (“forced circumstances”)	Peer pressure, economic necessity, sense of inevitability
Challenges in addressing early engagement in sexual activity	Cultural sensitivities, resource limitations, conflicting expectations (parents vs. educators)
Gender dynamics and power imbalances	Female vulnerability to exploitation, male pressure to prove masculinity, unequal power in sexual relationships
The intersection of individual and structural factors	Awareness of risks versus perceived inability to avoid them, impact of broader systemic issues on individual choices, limitations of knowledge-based interventions

These themes and sub-themes were derived from the extensive data gathered from classroom discussions with learners and teacher workshops. They represent recurrent processes and important ideas that could be used to analyse the complex phenomenon of early engagement in sexual activity by primary school learners in Musina.

The thematic structure captured the complexity of the topic to reveal how individual, interpersonal, community, and structural factors interact to produce the “forced circumstances” reported by the learners. Consequently, this analysis forms the basis for identifying obstacles both learners and teachers identified and highlights possible directions for intervention and future research.

## Results

This study explored the forced circumstances underlying early sexual debut among primary school learners in Musina, Limpopo. Although some findings reinforce existing knowledge, such as the role of poverty, peer pressure, and limited sex education, this research extends the field by presenting a nuanced, theory-informed analysis of how systemic inequality, adolescent agency, and culturally embedded norms intersect in a rural South African context. The integration of the Social-Ecological Model, Theory of Planned Behaviour (TPB), and Structural Violence allowed for a multidimensional interpretation of learners’ behaviours and perceptions. These frameworks were not only used to guide the data collection but also to structure the thematic interpretation and deepen our understanding of the underlying power dynamics, decision-making constraints, and social conditioning that shape early sexual behaviour.

### Environmental instability and structural violence: “We See It Everywhere”

Environmental deprivation emerged as a key enabler of early sexual socialisation. Learners reported a lack of physical privacy in their homes and exposure to sexual activity in public spaces. One 12-year-old girl shared:

At home, we all sleep in one room. Sometimes, I see things I should not. Moreover, in the community, people do not care. They do it [have sex] anywhere, even under trees where we can see. It makes it seem normal. (Learner – Vuyo, Grade 6)

This quote exemplifies how structural violence manifested in overcrowded housing and informal public spaces normalises sexual activity for children. Within the Social-Ecological Model, this reflects the interaction between the microsystem (family housing conditions) and macrosystem (urban design, poverty). The consistent exposure to adult sexual behaviours in community settings desensitises learners and fosters early normalisation of sex, weakening any perceived behavioural control (TPB) they might have over delaying sexual initiation.

### Socio-economic drivers and consumerist pressures: “It’s Not Just About Food”

While poverty is a well-established drivers of early sexual activity, this study reveals a complex layering of socio-economic aspiration, peer influence, and transactional sexuality among young learners. A 14-year-old girl explained:

Some girls go with Polo or DG6 drivers because they can buy us things from Temu or Shein. It is not just about food; we want to look good too. When you see your friends with nice hairstyles and clothes, you want that too. (Learner – Zodwa, Grade 7)

Zodwa’s account situates her sexual decision-making within a matrix of material desire, peer comparison, and structural exclusion from economic opportunity. Her statement reflects not only survival needs but also the internalisation of consumerist aspirations, revealing how adolescent self-worth is externally constructed through appearance and peer validation. This aligns with subjective norms and attitudinal pressure in TPB, while structural violence explains why learners must exchange sexual access for items that confer social legitimacy.

Moreover, a teacher noted:

Some girls intentionally get pregnant to access the child support grant. They see it as a way out of poverty, not realizing the long-term consequences. (Educator – Vusani)

Here, the perceived behavioural control (TPB) is again compromised not because adolescents fail to understand risks, but because systemic inequality limits alternative life strategies, compelling rational, though constrained, choices that prioritise economic stability over long-term well-being.

### Peer influence, masculinity, and identity: “To Be a Man. . .”

Peer norms emerged as both a motivator for early sexual debut and a form of identity validation, especially for boys. A 12-year-old boy recounted:

If you have not done it [had sex], your friends call you a baby. They say you are not cool. Sometimes you do it just to be part of the group, even if you are not ready. (Learner – Mpho, Grade 5)

Mpho’s response illustrates the subjective norms of the TPB: the need to conform to peer expectations supersedes personal readiness. Within the social-ecological framework, these influences stem from the mesosystem (peer interactions) and are amplified by the absence of counter-narratives in the school and home settings.

Similarly, a 13-year-old boy noted:

We believe that for you to be a real man, you must have sex with many girls, (Learner – Gundo, Grade 6)

Gundo’s quote exemplifies a hegemonic masculinity ideology, where sexual conquest is equated with male identity. These insights build on the literature but extend it by framing sexual activity not only as a peer norm but as a cultural rite of passage shaped by gendered power expectations a critical area for transformative intervention.

### Physiological maturation and education deficits: “They’re Left to Figure It Out”

Sexual maturation, while biologically driven, is poorly supported in the educational setting. A Life Orientation teacher explained:

We try to teach them about their bodies and the changes they are going through, but it is not enough. Some topics are considered taboo, so we skip them. The kids are left to figure things out on their own. (Educators – Khumbu)

This reinforces how incomplete sex education due to cultural taboos and teacher discomfort intersects with physiological development, leaving learners without the tools to manage emerging sexual impulses. The result is a disconnect between the intrapersonal level (biological change) and the institutional level (school education) in the Social-Ecological Model, leading to misinformed or risky choices.

### Parental pressures and normalisation of exploitation: “It’s a Way Out”

Parental endorsement of exploitative sexual relationships, often with older men, was reported as a coping mechanism in low-resource households. A teacher shared:

It is shocking, but some parents see these relationships as a way out of poverty. They tell their daughters, ‘If you can get a teacher or a truck driver, our lives will improve.’ It’s a cycle that’s hard to break. (Educator – Fhatu)

This reflects how the macrosystem (poverty, unemployment) in the Social-Ecological Model interacts with family-level decisions to reinforce structural violence. Families, often unintentionally, socialise children into seeing their bodies as economic assets, normalising transactional relationships as culturally acceptable and economically necessary.

### Gendered vulnerability and power imbalances: “It’s Hard to Say No”

The asymmetrical power dynamics between older men and adolescent girls underscore gendered vulnerability. One girl shared:

The blessers promise us things. They say they will take care of us and our families. When you are hungry and see no other way, it is hard to say no. (Learner – Linde, Grade 7)

Linde’s quote foregrounds the interaction between material deprivation, power imbalance, and coercive consent. This highlights structural violence in its purest form: adolescents do not “choose” but are compelled by constrained agency, shaped by hunger, inequality, and adult exploitation. Their inability to say no despite awareness of risk reflects a lack of real behavioural control, reinforcing the relevance of the TPB in settings of chronic vulnerability.

Overall, this study extends existing knowledge by showing how structural violence, poverty, and peer norms intersect to constrain adolescent agency and normalise early sexual activity. By applying the Theory of Planned Behaviour and the Social-Ecological Model, the findings reveal how individual choices are shaped by layered social and structural pressures, highlighting the need for multi-level, theory-informed interventions.

## Discussion

This study explored the drivers of early sexual debut among primary school learners in Musina, offering new insights into how socio-economic deprivation, peer influence, inadequate sex education, and gendered norms intersect in a rural South African context. While our findings align with existing literature,^9,43^ they also advance the field by applying an integrated theoretical lens to show how structural violence, behavioural intention, and environmental factors jointly erode adolescent agency.

### Socio-economic factors and structural violence

Participants references to “forced circumstances” highlight the material conditions of poverty, food insecurity, and lack of basic needs that shape early sexual decision-making. Galtung’s^35^ theory of structural violence offers a compelling lens to understand how these systemic inequities compel behaviour framed as choice but rooted in survival. Girls engaging in transactional relationships to secure goods or income demonstrate the erosion of autonomy under structural pressure, a finding consistent with Farmer’s^36^ work on poverty and vulnerability.

While previous studies have debated whether child support grants incentivise teenage pregnancy,^44^ our findings suggest a more nuanced reality. Adolescents perceive pregnancy as an economic strategy amid limited opportunities, which should be viewed less as a misuse of state welfare and more as a rational response to chronic poverty.^36^

### Environmental and cultural influences

Learners’ exposure to sexual activity within overcrowded homes and public spaces reflects how physical environments condition perceptions of normative behaviour. This supports Bronfenbrenner’s^32^ assertion that development occurs within layered environmental systems, where macrosystemic poverty filters into microsystem-level experiences. The prevalence of truck drivers and “blessers” in border communities such as Musina reinforces Delany-Moretlwe et al.^45^ findings on how transience and informal economies increase sexual vulnerability among adolescents.

Culturally, early sexual debut is often tolerated or even tacitly encouraged when it aligns with perceived survival or social advancement, creating challenges for public health messaging that relies on abstinence or moral framing.

### Peer influence and identity formation

Peer pressure emerged as a key subjective norm influencing early sexual behaviour, particularly among boys who equated masculinity with sexual conquest. This supports Ajzen’s^34^ Theory of Planned Behaviour, which posits that subjective norms, especially during identity-forming years, can override personal intentions or readiness. As Erikson^46^ noted, adolescence is a stage of identity consolidation, and learners are highly susceptible to norms that promise social inclusion.

Girls, on the other hand, were influenced more by peer comparison around appearance and consumption. This dual pressure masculinity through conquest, femininity through desirability, exacerbates gendered behavioural expectations and underscores the need for context-sensitive gender-transformative programming.^47^

### Educational factors and knowledge gaps

Inadequate and culturally restricted sex education remains a major barrier. Educators acknowledged skipping essential topics due to discomfort or fear of community backlash. This aligns with Kirby’s^9^ critiques of abstinence-only approaches and highlights the limitations of standardised curricula in diverse rural contexts. Without appropriate knowledge, learners are left to navigate physiological changes and sexual impulses with minimal guidance, undermining their capacity for informed decision-making and reducing perceived behavioural control.^34^

This disconnect also reflects a failure in the exosystem level of Bronfenbrenner’s model, where policy and educational content are not aligned with learners’ lived realities.

### Gender dynamics and power imbalances

The study affirms gender as a central axis shaping early sexual experiences. Female learners, in particular, reported coercion and economic dependence on older male partners. This dynamic reflects both gender-based violence and economic subordination, reinforcing the feminist perspective that links sexuality with broader power structures.^47^ Male learners, meanwhile, described social pressure to initiate sex early, often as proof of manhood.

These gendered scripts reinforce unequal power dynamics, normalise risk, and limit agency. Interventions must challenge these norms and offer alternative models of gender identity and empowerment.

### Parental influence and intergenerational cycles

Parental attitudes towards adolescent sexuality also played a significant role. In some cases, parents endorsed or facilitated exploitative relationships, viewing them as survival strategies. This reflects the long-standing entrenchment of poverty and intergenerational cycles of vulnerability. Rather than framing such practices as moral failures, they should be understood within the structural violence framework as rational choices constrained by systemic deprivation.^36^

Multi-level interventions that engage not just learners and educators but also caregivers are essential to disrupt these harmful cycles.

### Methodological reflections and study limitations

While the study offers critical insight, it is not without limitations. The use of group discussions may have introduced social desirability bias, with learners potentially adjusting their responses to appear acceptable to peers or facilitators. Although facilitators employed culturally sensitive strategies and ensured confidentiality, the sensitive nature of the topic remains a methodological challenge.^48^

Additionally, the purposive sampling of schools in Musina limits generalisability. While the use of multiple participant groups enhances trustworthiness, the absence of direct parental voices is a gap that future research should address.

### Actionable recommendations

The findings of this study highlight the complex interplay of socio-economic, cultural, and environmental factors contributing to early sexual engagement among primary school learners in Musina. Addressing these challenges requires a comprehensive, multi-sectoral approach that educates adolescents and transforms the broader social and economic conditions that shape their experiences. The following recommendations present targeted, context-specific strategies designed for the rural, resource-constrained setting of Musina, with special consideration of cultural sensitivity, educational realities, and structural limitations (Table 3).

**Table 3.** Study recommendations.

Recommendations	Actions
Implement multilevel interventions	Develop and implement integrated school and community programmes that combine health education, life skills, and psychosocial support.
	Involve school governing bodies, local clinics, NGOs, and faith-based organisations in co-delivering programmes to reinforce consistent messaging across environments.
Enhance economic empowerment initiatives	Launch vocational skills development workshops (e.g. sewing, beadwork, poultry farming) during school holidays to empower learners and their caregivers.
	Advocate for conditional cash transfer models linked to school attendance or health screenings to reduce reliance on transactional sex.
Revise and strengthen comprehensive sex education	Tailor sex education to the Musina context by including specific topics such as consent and refusal skills, understanding peer pressure, transactional sex, dealing with coercion, myths about fertility, gender equality, and puberty-related body changes.
	Use culturally appropriate language and examples familiar to the local Tshivenda-speaking population.
	Incorporate storytelling, role-plays, and locally developed visual aids to overcome literacy barriers.
	Train life orientation teachers annually using updated manuals co-developed with health professionals and community elders to reflect both evidence-based content and cultural appropriateness.
	Embed regular, safe discussion spaces (“Talking Circles”) within the Life Skills curriculum to encourage ongoing dialogue rather than one-off lessons.
Strengthen community engagement and awareness campaigns	Facilitate community dialogues involving parents, traditional leaders, religious groups, and local authorities to challenge harmful cultural norms and promote healthy adolescent development.
	Introduce “Parent-Learner Dialogue Days” where safe, guided intergenerational conversations can happen around sexual health and choices.
	Establish peer-led support groups and mentorship programmes to foster open discussions on sexual health and decision-making.
Promote gender-transformative approaches	Develop gender-sensitive interventions that address power imbalances and equip both boys and girls with the skills to challenge harmful gender norms.
	Integrate modules on healthy relationships, consent, and emotional literacy within the existing CSE.
	Incorporate gender analysis in adolescent programmes to ensure interventions foster equitable relationships and discourage coercion or exploitation.
Implement targeted interventions in high-risk areas	Prioritise schools and communities near the N1 trucking corridor and informal settlements for targeted sexual health outreach.
	Use mobile clinics to deliver health services such as contraceptive counselling, STI screening, and mental health support during scheduled school visits.
	Deploy youth-friendly peer educators from the local community to conduct after-school and weekend outreach.
Adopt adolescent-centred participatory approaches	Establish adolescent health committees in schools and link them to district-level youth advisory councils.
	Involve learners in co-designing IEC tools, campaign messages, and peer support activities to foster ownership and cultural resonance.
	Conduct annual adolescent-led community scorecards on service delivery to promote accountability and responsiveness.

### Implications for policy and practice

The study highlights the urgent need for systemic changes to address the structural drivers of early sexual debut among adolescents. Socio-economic disparities, gender power imbalances, and cultural norms contribute to an environment where young people face pressure to engage in risky sexual behaviour. Policies should address poverty’s root causes, challenge harmful societal norms, and improve access to education and healthcare. Furthermore, a multi-sectoral approach involving government, civil society, and local communities is essential to creating sustainable, long-term solutions that protect adolescents and promote their well-being.

### Limitations and future research

The findings of this study offer important insights into the multiple factors that influence early engagement in sexual activity among primary school learners in Musina. However, the study has limitations, as the possibility of social desirability bias cannot be discounted. Further longitudinal studies should therefore be conducted, and research conducted at other sites in South Africa could reveal both broad and place-sensitive phenomena.

## Conclusion

This study demonstrates that early sexual engagement among primary school learners in Musina is driven not by isolated personal choices but by a convergence of structural violence, socio-economic deprivation, peer norms, and inadequate sex education. By applying an integrated theoretical lens, the study reframes early sexual debut as a product of constrained agency shaped by poverty, gender inequality, and institutional neglect. To address these systemic drivers, multi-sectoral interventions are essential, combining comprehensive, culturally relevant sexuality education with socio-economic support and teacher training. Policymakers, educators, and community leaders must act collaboratively to dismantle the environmental and normative conditions that normalise risk and limit adolescent autonomy.

This research contributes to the growing recognition that adolescent sexual health must be addressed through structural reform, not solely behavioural change. Future studies should expand this work through longitudinal and cross-regional comparative research to understand the long-term and context-specific effects of socio-economic hardship. Mixed-methods approaches will further deepen understanding and support the development of targeted, evidence-based interventions that protect the health and potential of vulnerable youth in rural South Africa and beyond.
